# Master Regulators of Muscle Atrophy: Role of Costamere Components

**DOI:** 10.3390/cells10010061

**Published:** 2021-01-03

**Authors:** Luisa Gorza, Matteo Sorge, Laura Seclì, Mara Brancaccio

**Affiliations:** 1 Department of Biomedical Sciences, University of Padova, 35121 Padova, Italy; 2 Department of Molecular Biotechnology and Health Sciences, University of Torino, 10126 Torino, Italy; matteo.sorge@unito.it (M.S.); laura.secli@unito.it (L.S.); mara.brancaccio@unito.it (M.B.)

**Keywords:** costamere, muscle atrophy, nNOS, melusin, atrogene, dystrophin, muscle disuse, cachexia, sarcopenia, aging

## Abstract

The loss of muscle mass and force characterizes muscle atrophy in several different conditions, which share the expression of atrogenes and the activation of their transcriptional regulators. However, attempts to antagonize muscle atrophy development in different experimental contexts by targeting contributors to the atrogene pathway showed partial effects in most cases. Other master regulators might independently contribute to muscle atrophy, as suggested by our recent evidence about the co-requirement of the muscle-specific chaperone protein melusin to inhibit unloading muscle atrophy development. Furthermore, melusin and other muscle mass regulators, such as nNOS, belong to costameres, the macromolecular complexes that connect sarcolemma to myofibrils and to the extracellular matrix, in correspondence with specific sarcomeric sites. Costameres sense a mechanical load and transduce it both as lateral force and biochemical signals. Recent evidence further broadens this classic view, by revealing the crucial participation of costameres in a sarcolemmal “signaling hub” integrating mechanical and humoral stimuli, where mechanical signals are coupled with insulin and/or insulin-like growth factor stimulation to regulate muscle mass. Therefore, this review aims to enucleate available evidence concerning the early involvement of costamere components and additional putative master regulators in the development of major types of muscle atrophy.

## 1. Introduction

Skeletal muscle atrophy represents a pathological outcome rather widespread and consequent to several conditionns, among which are aging, bedding, muscle inactivity (with or without loss of innervation), systemic diseases like heart failure, cancer, respiratory insufficiency, and obesity, not to mention genetic diseases affecting skeletal myofibers. Although muscle atrophy is characterized by the loss of muscle mass and force, it develops in each of the above listed conditions with specific signatures [1], whose features cannot be separated from a comprehensive mechanistic view.

Loading is one major stimulus for muscle work and growth, and reduced muscle load, such as it occurs during prolonged bed-rest or immobilization, is a powerful driver for atrophy development, especially in anti-gravitational muscles such as limb muscles (soleus and vastus lateralis) [2]. Muscle mass loss is fast (about 18% in a month in a young human, the same amount in a week in an old one) [3], mostly due to myofiber atrophy, which involves all the fiber type populations, and is accompanied by a slow-to-fast fiber type transition, as recently reviewed by Vikne et al. [4].

Motoneuron activity represents the other major regulator of muscle mass. Loss of stimulation from either primary or pyramidal motoneuron quickly induces severe muscle atrophy, which results from both loss of myofiber nuclei, secondary to increased apoptosis, and size, and predominantly affects fast myofibers in a context where a slow-to-fast fiber type transition occurs. Indeed, muscle response to the loss of neuronal innervation apparently depends on its fiber type composition, since each fiber type shows marked difference in proteome remodeling [5].

Age-related muscle wasting (sarcopenia) leads not only to a decline in performance and fitness of elderly people, but also to an increased risk of falls and progressive loss of functional independence in daily activities [6,7]. Sarcopenia implies a gradual loss of muscle strength, which starts in humans after the age of 60, often preceding the decrease in myofiber number and size, and preferentially involving the fast fiber population [8]. The European Working Group on Sarcopenia in Older People defines sarcopenia by the presence of low appendicular lean mass/height^2^ and altered muscle strength, relative to sex-specific threshold values, and presarcopenia by the presence of decreased muscle mass without impact on muscle force development and contraction [9]. The key difference between disuse atrophy and sarcopenia is the presence of a decrease in size and number of muscle fibers in the latter, whereas the former involves a decrease in fiber size only [10].

Cachexia is a multifactorial syndrome characterized by systemic inflammation, skeletal muscle atrophy, adipose tissue wasting and metabolic abnormalities, leading to multiorgan dysfunction. Different pathologies such as cancer, heart failure, and respiratory insufficiency may trigger this complex manifestation that is often responsible for reducing quality of life and worsening disease outcomes [11]. Cachexia-related muscle atrophy cannot be reversed by increasing nutrition, suggesting that important metabolic alterations are occurring in the patients. Systemic inflammation, and specifically the prolonged presence of cytokines in the circulation, has been recognized to play a causal role in this context [12,13,14].

Cancer cachexia and systemic diseases often lead to asthenia, where the loss of muscle force and muscle weakness is independent from muscle mass loss. Asthenia has been recently recognized as a relevant clinical condition, which is influenced by patient’s subjectivity and difficult to diagnose [13]. Pathogenesis of asthenia at the myofiber level remains obscure, although a reduction in muscle strength, which is not explained by a loss in muscle mass, has been already observed during aging [15].

The pioneering studies from Bodine’s and Goldberg’s laboratories [16,17] identified key genes, whose upregulation is shared in every of these conditions and leads to muscle atrophy development (the so-called atrogenes). Although atrogenes are involved in muscle protein catabolism, they represent a minor shared component of the broad transcriptome change accompanying skeletal muscle atrophy development in each of the described conditions [18]. Several additional players do contribute, and still unanswered major questions concern whether they simply enhance atrogene upregulation or play independent and specific key roles in their regulation. Another currently unsolved aspect is the identification of atrophy initiators, namely the sensors, which activate/deactivate signaling pathways leading to gene expression and atrophy development. The identification of sensors implies taking into account muscle fiber structure, organelles and their anatomical relationships. Among these, the costamere represents a major muscle multiprotein complex, which co-ordinates myofibril contraction with sarcolemma and the extracellular matrix. Therefore, the aim of this review is to present current evidence about the involvement of master regulators and sensor candidates in the different conditions leading to muscle atrophy, focusing on the contribution of key components of costamere structure and function.

## 2. Master Regulators of Muscle Atrophy

Definition of a master regulator implies its requirement, yet absolute or complementary, to initiate a biological/pathological process. Experimental models reproducing different conditions leading to muscle atrophy have been developed and used to identify master regulators of atrophy by means of either pharmacological or genetic tools. Detailed reviews about the involvement of regulators of muscle transcription, protein synthesis, and anabolic signaling pathways, protein catabolism and autophagy have recently appeared [19,20,21,22]. Therefore, we would largely refer to these contributions and limit our presentation to knowledge concerning evidence of the major or partial involvement of these regulators with costamere components in different atrophy conditions.

### 2.1. Transcriptional Regulators of Atrogenes

Atrogenes, i.e. genes codifying for E3 ubiquitin ligases upregulated during muscle atrophy, are targets of several transcription factors, which act directly and independently, although co-operative regulation has been also detected [16].

#### 2.1.1. FoxOs

FoxOs (FoxO1, FoxO3, FoxO4) are involved in most types of muscle atrophy, since the inhibition or genetic deletion of multiple FoxOs completely prevented its development induced by fasting, glucocorticoid treatment and diabetes [19]. Conversely, deletion of all FoxO family members only attenuated muscle atrophy induced by limb immobilization and reduced, but not abolished, the expression of major atrogenes, such as MuRF1 and MAFbx/Atrogin-1 [23]. A comparable partial effect occurred for denervation-induced atrophy in mice with multiple FoxO KO [24]. Furthermore, FoxO apparently does not play a role in sarcopenia development [25], whereas MuRF1 is involved, since its deletion resulted in the maintenance of muscle mass with age [26]. Activation of FoxO3 results from the deregulation of several signaling systems, among which are: (i) the derangement of the Insulin Receptor (IR) cascade [19], (ii) the untethering of the neuronal isoform of Nitric Oxide Synthase (nNOS) from sarcolemma [27,28,29,30], (iii) the excessive activation of the anabolic response [19], possibly secondary to the lack of AMPK activation [20,31], (iv) the loss of FoxO3 inhibitory posttranslational modifications by means of HDAC1 [32,33], and (v) the inhibition of Akt activity by Smad2/3 signaling [34].

#### 2.1.2. NF-κB

The family of NF-κB transcription factors consists of five members, all expressed in skeletal muscle, RelA/p65, c-Rel, RelB, NF-κB1 (p50), and NF-κB2 (p52), forming homo- and heterodimers. In the absence of specific stimuli, these dimers are bound to IκBs (inhibitors of NF-κB) and kept transcriptionally inactive. The activation of the NF-κB “canonical pathway” induces the formation of the IKK (IκB kinase) protein complex containing the IKKα and IKKβ kinases and the regulatory subunit IKKγ. The activated IKK complex phosphorylates IκB inducing its detachment from NF-κB and its degradation. As a result, NF-κB dimers can enter the nucleus and regulate the transcription of a number of genes mainly coding for immunoreceptors, acute phase proteins and cytokines. The non-canonical activation of the NF-κB pathway is mediated by a specific group of stimuli, including ligands of a subset of tumor necrosis factor receptor (TNFR) superfamily members, such as LTβR, BAFFR, CD40, and RANK. The engagement of these receptors activates the NF-κB inducing kinase (NIK) that phosphorylates and activates IKKα. In turn, IKKα phosphorylates p100 stimulating its cleavage and the generation of p52 that together with RelB enters the nucleus to regulate gene transcription [35].

NF-κB activation increases dramatically in muscles during unloading, denervation, cachexia and sarcopenia and the inhibition of the NF-κB pathway has been shown to protect myofibers from atrophy in different animal models [36,37,38,39,40,41,42,43].

Both the canonical and the alternative NF-κB pathways play a role in atrophy induced by unloading [38,43,44,45] and the inhibition of IKKα or IKKβ activity, through the overexpression of dominant negative enzymes, robustly inhibits atrophy induced by 7 d of unloading in rats [45,46]. Nevertheless, in vivo treatments with an IKKβ inhibitor failed to inhibit expression of MuRF1 and MAFbx/Atrogin-1 after 3 d of unloading [47].

The involvement of the canonical NF-κB pathway in muscle mass regulation has been also demonstrated using mouse genetic models. Indeed, transgenic mice expressing constitutively active IKKβ selectively in muscles, appear normal at birth, but rapidly develop chronic atrophy characterized by severe muscle wasting [40]. This phenotype is rescued by the concomitant expression of the IκB supersuppressor, a mutant form of IκB resistant to degradation, able to inhibit NF-κB activation even in presence of IKKβ activation [40]. It has been demonstrated that, in response to sciatic nerve resection, NF-κB activity is increased by nine-fold after 14 d, when muscle mass has already decreased by 60%. However, the fact that transgenic expression of the IκB supersuppressor can partially rescue muscle mass and myofiber cross-sectional area indicates a contribution of NF-κB activity in denervation-induced atrophy [40]. In fact, in skeletal muscles of IKKβ conditional null mice denervation-induced atrophy is strongly reduced and the characteristic shift of fibers toward a fast phenotype is impaired [41]. On the other hand, in a mouse model of tumor-induced cachexia, muscle NF-κB activity was upregulated by six-fold after 12 d from cancer cell injection, i.e., simultaneously with myofiber atrophy appearance. The expression of the IκB supersuppressor showed a relevant effect in inhibiting muscle wasting and prolonging mice survival, in the absence of alterations in tumor growth [40]. 

The activation of the NF-κB pathway in skeletal muscle atrophy is mainly due to the binding of cytokines on muscle surface receptors [42]. The ability of IL-1, TNF-α, and TNF-related weak inducer of apoptosis (TWEAK) to promote skeletal muscle atrophy has been proved in vitro and in vivo [48,49]. These cytokines, either released at distant sites, in the case of tumor-induced cachexia [50], or locally, from skeletal muscle and neighboring tissues, in denervation- and disuse-induced atrophy [51,52], activate the NF-κB pathway, fostering NF-κB activity and cytokine production and generating a vicious circle. NF-κB activation in skeletal muscle has also been found directly responsible for inducing the expression of the ubiquitin ligase MuRF-1 [41] and for negatively regulating *MyoD* gene expression [39].

#### 2.1.3. Smad3

Smad transcription factors are activated by myostatin (a member of the TGF-β superfamily) and are potent inducers of MAFbx promoter activity [16,34].

Increased myostatin availability usually follows inflammatory conditions and extracellular matrix remodeling, such as those occurring in cachexia, especially secondary to systemic inflammatory diseases, and during aging [53]. Myostatin negatively regulates Akt activation, enhancing atrogene expression. However, myostatin contribution appears dispensable in the development of muscle unloading atrophy [54]. On the other hand, myostatin is a negative regulator of satellite cell proliferation and commitment to differentiation. Increased myostatin signaling has been hypothesized to play a major role in sarcopenia development [34], although no apparent increase in myostatin levels affects sarcopenic humans [25]. Myostatin plays a role also in cancer cachexia, where it impairs muscle mass regulation via p53 and p21 upregulation [55].

#### 2.1.4. ATF4

ATF4 is a transcription factor that binds to the cAMP response element and acts as a master transcription factor for adaptation to various stress, such as endoplasmic reticulum (ER) stress, amino acid starvation, mitochondrial stress or oxidative stress. ATF4 protein synthesis increases in response to eIF-2-alpha phosphorylation consequent to PERK activation, and regulates gene expression of the transcription factor CHOP [56].

ATF4 is upregulated already after 3 d of muscle immobilization and increased ATF4 expression is sufficient to induce atrophy of fast-type 2 fibers, by up-regulating the transcription of the cell-cycle inhibitor p21, and MuRF1 and MAFbx atrogenes [57]. Although the involvement of p21 up-regulation in several muscle atrophy types still awaits investigations to explore possible additional functions [16], a lower number of muscle nuclei (both satellite cell and true myonuclei) and lower BrdU incorporation characterize rat soleus muscle after denervation, implying reduced mitotic activity, in addition to myonuclei loss [58]. Muscle-specific ATF4-KO mice are partially and transiently resistant to immobilization-induced muscle atrophy, but, strikingly, they did not exhibit muscle sparing following denervation [57]. This latter feature appears surprising, since ER-stress response activation is a relevant component of muscle atrophy development after denervation and in cancer cachexia [21,59], in addition to other muscle disorders [60]. Strikingly, the inhibition of ER stress with the chemical chaperone 4-PBA not only led to accelerated muscle loss in lung cancer-bearing mice, but also to significant muscle atrophy in naïve mice [21]. Indeed, the ER-stress response plays a relevant role in the regulation of the muscle mass, being involved in its maintenance in cancer cachexia and during muscle maturation [21,61]. Such a complex contribution derives also from the peculiar role played in skeletal muscle by some of the effectors of the ER-stress response, such as the Glucose-regulated protein Grp94/gp96 chaperone, the Hsp90 ER-paralog. Grp94/gp96 not only has muscle-required growth factors (GF), like Insulin-like GF-I and -II, and pro-insulin, as exclusive clients for folding [62], but also interacts with several non-client proteins, among which nNOS (see Section 2.2.2) and the Heregulin Receptor HER2, dictating their subcellular distribution [63].

#### 2.1.5. p53

The transcription factor p53 is well-known for its role in the preservation of genome stability, as oncosuppressor, and in the promotion of the apoptotic response.

Different stimuli (unloading, denervation, aging) increase expression of p53 and target genes in skeletal muscle, suggesting an important role in atrophy development [64,65,66,67,68,69,70,71]. In hindlimb unloading, p53 expression starts to increase within 1 d of immobilization, before muscle atrophy onset [57]. Increased p53 expression is partially responsible for the fiber atrophy induced by immobilization, by acting independently from the other pro-atrophic regulator ATF4 on p21 expression [57]. Indeed, p21 is highly expressed in adult skeletal muscle fibers during a wide variety of atrophy conditions, including muscle disuse, fasting, aging, and systemic diseases [72]. The exact mechanism by which p53 induces atrophy is still controversial. One possibility is that p53 reduces muscle mass by increasing the loss of myonuclei by means of apoptosis. Indeed, evidence of increased p53 expression and apoptotic index have been provided for the soleus muscle after 48 h of hindlimb suspension [73]. Similarly, p53 protein content is markedly elevated in parallel with the upregulation of Bax, in rat gastrocnemius muscle after 14 d of denervation [67]. In sarcopenia the exact role of p53 is debated. Some reports suggest that chronic activation of p53 leads to premature myofiber aging associated with a significant atrophy [74,75]. This is confirmed by some evidence demonstrating that p53 is higher in older muscle tissue and regulates sarcopenia [64]. Another study suggests that p53, by binding directly to the myogenin promoter, can repress its transcription, impairing the maintenance of muscle tissue homeostasis [71]. Another theory suggests that nNOS controls p53 inactivation by means of S-nitrosylation. In muscle aging, the altered shuttle of nNOS to the nucleoskeleton [76] determines a fail in p53 S-nitrosylation, which results in MuRF-1 gene expression upregulation [77]. Consistently, p53-null mice are prone to cancer development but resistant to cancer-induced muscle atrophy [74]. In line, the muscle wasting secondary to radiation therapy can be blocked by chemical inhibition of p53 [78]. In TNFα-induced cachexia, p53, in concert with its target gene *PW1*, plays a role in blocking muscle differentiation [74]. Similarly, in doxorubicin-induced muscle atrophy p53 exerts its effect via *PW1* [74]. The expression of p53 affects differently fiber types in tumor-induced cachexia. Indeed, the loss in fast fiber size is reduced markedly in p53 null mice. Conversely, the loss of p53 induces only a mild effect in slow fibers [74].

#### 2.1.6. Hippo Pathway

The Hippo pathway, by means of the MST1-kinase cascade, negatively regulates the activation of YAP/TAZ, and cell proliferation and apoptosis in organ development [22]. In the skeletal muscle, YAP positively regulates basal skeletal muscle mass and protein synthesis.

Loss of muscle innervation activates the Hippo pathway and the inhibition of MST1 is sufficient to prevent atrophy in denervated, fast-twitch muscles [79]. Conversely, but in parallel, denervation increases YAP protein amount and activity in myonuclei, as a compensatory pro-trophic signal to attenuate muscle atrophy development [80]. YAP/TAZ positively regulate satellite cell/myoblast activation, and we tentatively speculate that dysfunctions in this pathway might play a relevant role in muscle atrophy development, especially in sarcopenia, where reduced recruitment of satellite cells appears to be mechanistically involved in loss of muscle mass [81]. However, such a hypothesis needs to be confirmed by further extensive investigations, especially in the light of a recent report about the pro-atrophic role played by YAP in a genetic model of sarcopenia [82].

### 2.2. Oxidative and Nitrosative Stress

Oxidative stress, together with nitrosative stress, represents a major player of muscle atrophy development. Systemic inflammation or diseases accompanied by inflammatory responses, such as heart failure, respiratory insufficiency and cancer, obviously account for higher levels of diffuse oxidative stress. Conversely, its increase during muscle disuse, such as following denervation or immobilization, remains still to be fully explained, since oxidative stress represents a relevant byproduct of muscle activity [83,84].

Increased oxidative stress in the inactive muscle derives from the imbalance between the muscle anti-oxidant defense, reduced by the increase of protein catabolism, and the physiological oxidant production [85]. However, the upregulation of chaperones and enzymes involved in the anti-oxidant defense occurs before muscle atrophy development, supporting the hypothesis that the increase in oxidant production anticipates the increase in protein catabolism [86,87]. Available evidence concerns increased accumulation of oxidative modifications, such as the presence of protein covalent adducts (carbonylation, binding of the 4-hydroxyl-nonenal moiety, tyrosine nitrosylation, advanced glycation end products) or oxidized species (disulphide bond covalent species) by means of biochemical/immunochemical assays [30,86,88,89,90,91]. Indeed, unbiased direct demonstration of increased oxidant accumulation requires specific approaches/probes and use of intact muscle or isolated myofibers [83,92]. The hypothesis that oxidative stress, like calcium ions, may rise and act locally, as well as globally, in the muscle fibers to initiate signaling in distinct subcellular compartments [84] should be also taken into account when discussing either positive or negative findings.

#### 2.2.1. Reactive Oxygen Species (ROS)

Putative sources of increased ROS production in skeletal muscle have been extensively reviewed [85,93] and identified in mitochondria and NADPH-oxidases, by means of the use of specific inhibitors. NADPH-oxidases appear the major source of cytosolic ROS during acute exercise bouts and are involved in exercise-induced changes, such as the translocation of the glucose transporter 4 (GLUT-4) and the activation of pro-trophic pathways [84]. Mitochondrial ROS have been suggested to increase post-exercise only, i.e., at rest [84]. Indeed, dysfunctional mitochondria characterize long periods of muscle inactivity [93] and both dysregulation of mitochondria fusion and alteration in structure and function negatively affect muscle mass in several conditions [94]. Skeletal muscle atrophy, secondary to unloading, denervation, fasting or FoxO3 overexpression, relieves after counteracting the downregulation of PGC-1α, the master regulator for mitochondrial biogenesis [95,96]. Therefore, reduced mitochondrial renewal might explain the organelle deterioration and its decrease in respiratory capacity. 

Additional possible mechanisms leading to increased mitochondrial ROS production after prolonged muscle unloading/inactivity were identified by changes in cardiolipin amount and composition [97], or increased mitochondrial Ca^2+^ secondary to ryanodine receptor 1 (RYR1) leakage [98]. On the other hand, only a few studies concerned the early involvement of mitochondrial dysfunction in atrophy development by unloading/inactivity. Mitochondria-targeted antioxidants, such as SS-31 and MitoTEMPO [99], were administered to laboratory rats before exposure to either a 12 h bout of mechanical ventilation [88] or a 6 h bout of hindlimb unloading [30]. In both cases, scavengers blunted the increase in oxidatively modified proteins and hampered the signaling required for atrophy progression, in the former case by reducing atrogene expression [88], and, in the latter one, by counteracting the loss of active nNOS from sarcolemma, an event which induces FoxO3 nuclear translocation [30]. Interestingly, SS-31 targets cardiolipin, optimizing mitochondrial electron transport and ATP synthesis [100], but changes in cardiolipin amount and composition after short exposure to muscle inactivity have not been investigated yet. The requirement of mitochondrial ROS production was apparently ruled out by the recent finding that transgenic mice expressing catalase in mitochondria are not protected from atrophy induced by 7 d-unloading [101], at variance with other reports where exogenous catalase expression or administration of mimetics [89,102] appeared effective in attenuating it. As recognized by Authors themselves, such a discrepancy might be explained either by experimental differences (recombinant mitochondrial-directed catalase is effective against muscle atrophy of the unloaded rat [102]) or by the major involvement of superoxide anion, the hydrogen peroxide precursor.

Although increased hydrogen peroxide production from muscle mitochondria has been detected only 3–5 d after transection of the peroneal nerve or the sciatic nerve, respectively [103,104], a recent extensive transcriptomic analysis, performed at different times after sciatectomy, indicates a very early role for oxidative stress in denervation-induced muscle atrophy [87]. Increased transcript accumulation for genes involved in calcium release, oxidoreductase activity and antioxidant activity, occurs between 30 min and 12 h after sciatectomy, compared to controls that underwent surgery without denervation. Interestingly, cytochrome P450 appeared among the most activated signaling pathways, suggesting endosomes as the preferential early ROS source in the denervated myofiber [87].

#### 2.2.2. Reactive Nitrogen Species (RNS) 

The contribution of NO, NOS and nitrosative stress to muscle atrophy development is still controversial, despite of the abundant evidence concerning their involvement in muscle atrophy [20,90,105,106]. A major point of disagreement concerns the actual availability of NO during muscle atrophy development. It has to be mentioned that adequate measurements of NO require isolated muscle and use of spin-trap electron paramagnetic resonance [27,107,108]. Opposite results were obtained with such a technique in the soleus muscle after 7d-unloading [27,109]. As suggested by Sharlo et al. [109], a possible explanation for this discrepancy derives from methodological aspects (use of intact frozen muscles [109] vs minced muscle maintained at room temperature [27]). Unfortunately, this approach does not discriminate between endothelial and myofiber NO sources. 

**Figure 1:**
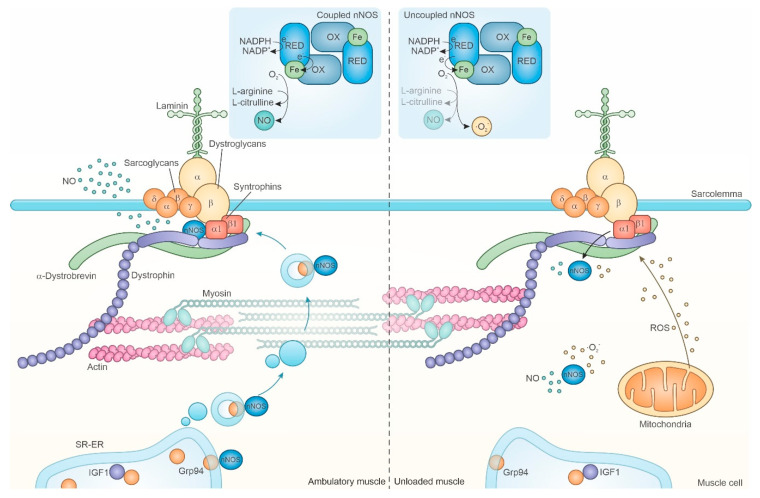
The neuronal NOSμ isoform interacts with the Grp94/gp96 chaperone and is delivered at the subsarcolemma by docking at the DCG. Unloading-induced mitochondrial ROS production causes nNOSμ untethering from DGC and translocation in the sarcoplasm, where the enzyme through either “coupled” or “uncoupled” NADPH oxidation (inset) leads to NO/O_2_^−^ production, respectively, and FoxO3 activation. NO = nitric oxide; nNOS = neuronal nitric oxide synthase; SR-ER = sarco-endoplasmic reticulum; IGF1 = insulin-like growth factor 1.

In the skeletal myofibers, NO is synthesized by the neuronal nNOSμ isoform that localizes at sarcolemma by tethering to the dystrophin glycoprotein complex (DGC) (Figure 1). At present, no controversy exists about the redistribution of the nNOSμ isoform from sarcolemma to sarcoplasm after exposure to unloading or denervation [27,30,89,110,111], whereas contrasting reports concern actual enzyme and NO levels [27,107,108,109,111,112]. The possibility that variable atrophy degree and/or duration of denervation/unloading affected nNOS levels was explored by extensive transcriptomic and proteomic analyses in rat soleus muscle after different unloading times (from 6 h to 7 d) [30]. Results showed an early and severe, but transient, decrease of both nNOS mRNA and protein, which returned at physiological levels after about 7 d-unloading, however, without the recovery of the physiological subcellular localization at sarcolemma [27,28,30,89]. Consequently, NO production in myofibers is expected to vary during unloading, because of changes in the enzyme amount, in addition to the site of production [20,105,113].

The beneficial pro-trophic effects of NO are largely recognized [113] acting on pro-trophic signaling [106]. Exogenous administration of NO-donors or L-arginine was indeed effective in attenuating unloading-induced muscle atrophy [107,114] and the drop of satellite cell proliferation [114]. However, the same effects were observed also in the dystrophic muscle [107,115] which expresses very low levels of nNOS, arguing whether another NOS isoform (the endothelial one?) might be involved in this response. Alternatively, L-arginine might act upon and stabilize those nNOS molecules still tethered at sarcolemma [107]. However, such a possibility has not been investigated yet. Conversely, misplaced sarcoplasmic NO production has been hypothesized to lead to unwanted consequences, first of all to FoxO3 activation, as showed by overexpressing nNOS in cultured myotubes [27]. Since maturation of the DGC requires more than 7 d growth in differentiation media, the overexpressed nNOS enzyme localizes meanwhile exclusively in the sarcoplasm. Comparably, in the absence of dystrophin, i.e., in the dystrophic muscle, the extremely low level of sarcoplasmic nNOSμ is nevertheless responsible for decreased muscle performance, which ameliorated after the expression of a mini-dystrophin construct and enzyme docking at sarcolemma [116] or of a palmitoylated nNOSμ that tethers directly at sarcolemma [117]. It is relevant to recall that any beneficial effect consequent to the sarcoplasmic overexpression of the less active nNOSα isoform did not involve the myofibers at all, but only reduced the population of M2 macrophages and the degree of fibrosis [118]. 

In the unloaded soleus muscle, the knocking-out of nNOS gene or the inhibition of its activity attenuated FoxO3 activation and muscle atrophy [27]. Silencing of nNOS mRNA before a 6-h unloading bout, abolished FoxO3 accumulation in myonuclei [30]. The same effect, concomitantly with the attenuation of muscle atrophy, occurred when physiological muscle levels of the Grp94 chaperone, which interacts with nNOS mostly in the sarcoplasmic reticulum (SR)/ER, were specifically maintained during unloading by means of gene transfer or pharmacological treatment [28,29]. Blunting of FoxO3 nuclear accumulation, and muscle atrophy attenuation, required physiological levels of Grp94 that operated by targeting nNOS to sarcolemma [28,29]. In a comparable way, Grp94 is required to target HER2 molecules to cancer cell plasmalemma and improve viability [119].

In addition to generate regional changes in NO production, the redistribution of nNOS to the sarcoplasm might facilitate an “uncoupled” NADPH oxidation (uncoupled from NO formation), decreasing NO production and generating superoxide anion [120] (Figure 1, inset). Neuronal NOS has a particular propensity to catalyze this “uncoupled” reaction. Furthermore, because of the very rapid reaction of superoxide with NO, the synthesis of both species by the same enzyme, which exists as a dimer, is likely to result in peroxynitrite formation [120], fostering nitrosative stress. The hypothetical accumulation of “uncoupled” active nNOS molecules in the sarcoplasm would be consistent with both the evidence of reduced NO production in unloaded muscles [107,108,109] and the requirement of an active sarcoplasmic nNOS to activate FoxO [27,30]. Indeed, histochemistry for NADPH-diaphorase [30,121], which is widely used to demonstrate the subcellular localization of active nNOS molecules, detects only the activation of the carboxy-terminal reductase domain, which acts upstream and provides electrons to the NO-generating oxidase domain in the “coupled” conformation, or directly to O_2_ when “uncoupled” [120].

### 2.3. Mechanotransduction

**Figure 2:**
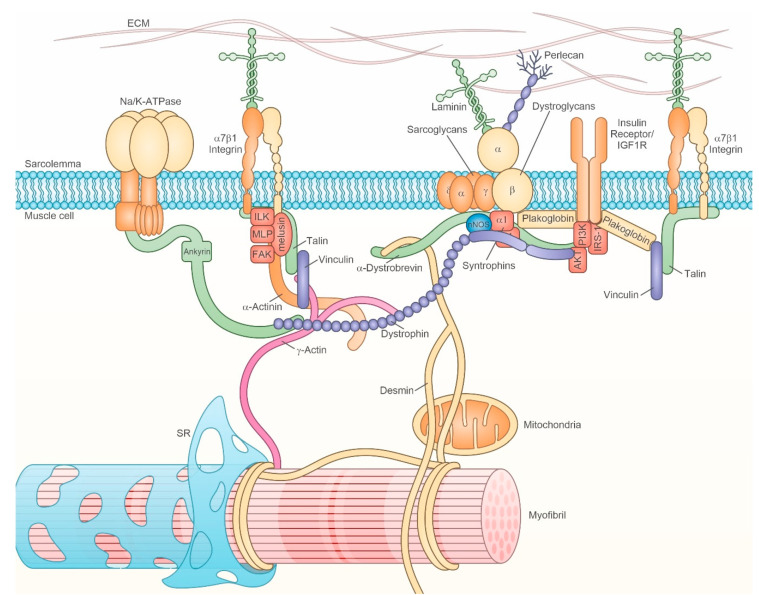
The sarcolemmal costamere components and their interactors form a supramolecular platform specialized in mechanostransduction and signal integration (only a part of the components is shown in the figure). ECM = extracellular matrix; ILK = integrin-linked kinase; MLP = muscle LIM protein; FAK = focal adhesion kinase; nNOS = neuronal nitric oxide synthase; PI3K = phosphoinositide 3-kinase IRS-1 = insulin receptor substrate-1; IGF1R =insulin-like growth factor 1 receptor; SR = sarcoplasmic reticulum.

Major determinants of muscle activity are the neuromuscular junction (NMJ) and the ability to sense mechanical stretch through costameres, i.e., multiprotein complexes that function as mechanotransducers, transforming mechanical load in biochemical signals, which, in turn, trigger specific responses in terms of gene expression, protein synthesis and organization. Skeletal muscle expresses a number of mechanotransducers with different sensitivity and specific responses to tension. Costameres align between the sarcolemma and underlying sarcomeric myofibrils coincidently with Z-discs and M-lines, and connecting them to the extracellular matrix (ECM) [122,123,124]. Although a high number of costamere components belongs to the cytoskeleton, relevant members, such as the DGC, integrins and ionic pumps/channels, localize at the sarcolemma [124] (Figure 2). Components of the DGC are essential for mechanoprotection from shear stress and reduce contraction-induced injury [125]. Integrins collect forces spreading laterally to the long axis of the sarcomere, from each myofibril to the neighboring one, and channel them across the sarcolemma to the extracellular matrix, by providing up to 70% of the muscle contraction force [123]. Costamere proteome shows a fiber-type specialization, which appears to be involved in dictating sarcomere composition during resting, loading and after pharmacological immobilization with botulin toxin [126,127].

In addition to nNOS, which is a component of DGC, recent investigations provided relevant and further evidence of the regulatory role of costamere components on muscle mass [128,129]. Our laboratories demonstrated the requirement of the integrin-binding, chaperone protein melusin to counteract muscle disuse atrophy [128], whereas another report identified plakoglobin as the mediator of physical and functional interaction between DGC and the Insulin receptor (IR) [129]. These and previous pieces of evidence further amplify the concept of a costamere as more inclusive, where a sovramolecular complex hosting different protein–protein interactions serves as a “signaling hub”, as dubbed by Eid Mutlak et al. [129], to regulate myofiber size.

#### 2.3.1. Dystrophin Glycoprotein Complex (DGC)

Dystrophin, sarcoglycans, dystroglycans, syntrophins are major components of the DGC, which hosts several others relevant regulators, such as nNOS and the recently identified interactor plakoglobin [129] (see the Section 2.3.3), and works, together with integrins, to provide a tight connection between the sarcomere and ECM components like laminin and the heparan sulfate perlecan [15,130,131,132,133]. At the core of the DGC is dystrophin, a large 427-kDa protein, which interacts with actin filaments at its amino terminus and connects to the sarcolemma by binding to β-dystroglycan and α1-syntrophin at its carboxyl end.

Among the conditions leading to muscle atrophy, loss of dystrophin usually occurs as a late event, probably because of the extreme long life of this protein [134]. In aged muscle, dystrophin loss preferentially affects flexor muscles and is accompanied by increased amount of other DGC and costamere components, such as β-dystroglycan, α-sarcoglycan, sarcospan, desmin and muscle LIM protein [135]. Conversely, reduced dystrophin protein levels, but not transcript ones, represent an early event in cachexia development, since they occurred before the reduction in mean fiber diameter [136]. A loss of dystrophin is accompanied by hyperglycosylation of β-dystroglycan and β-sarcoglycan, and by a loss of interactions between β-dystroglycan and α-dystroglycan, or dystrophin, without overt changes in the subcellular localization of the former proteins. Dystrophin overexpression in mice counteracted cancer-induced muscle loss and atrogene expression, indicating DGC disruption as a major switch of cachexia development [136]. Interestingly, insulin resistance develops during cachexia, such as in the presence of muscle dystrophy or other types of muscle atrophy, enclosed that one accompanying obesity [137]. Indeed, as will be reviewed in Section 2.3.3, the DGC appears to be connected physically and functionally to the IR by means of β-dystroglycan binding to plakoglobin [129]. Therefore, DGC deregulation influences IR signaling and the way around.

**Figure 3:**
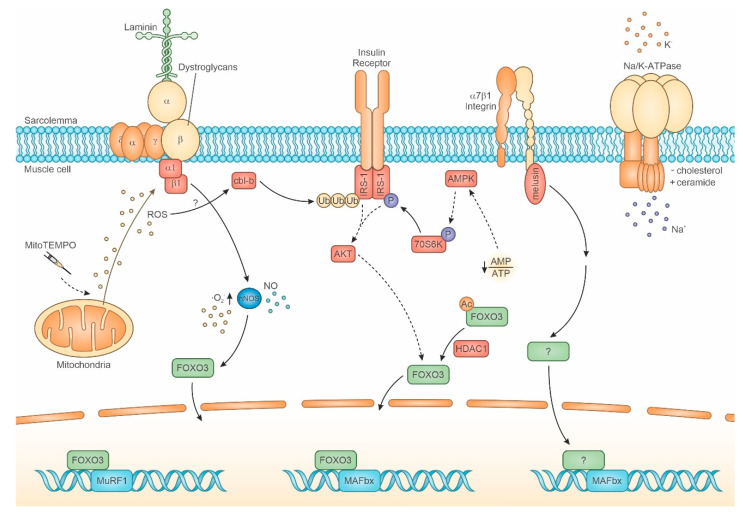
Signaling pathways activated after a 6–12 h bout of muscle unloading in costameres. Continuous lines indicate stimulatory effects, while discontinuous lines indicate inhibitory effects. Cbl-b = Casitas B-lineage lymphoma-b ubiquitin ligase; Ub = ubiquitin; nNOS = neuronal nitric oxide synthase; FOXO3 = forkhead box O3; MuRF1 = muscle RING-finger protein-1; MAFbx = muscle atrophy F-box; HDAC1 = histone deacetylase 1; Ac = acetylation; IRS-1 = insulin receptor substrate-1; 70S6K = Ribosomal protein S6 kinase p70; P = phosphorylation; AMPK = AMP-activated protein kinase.

Indeed, in the absence of an early loss of dystrophin, other events, such as the unloading- and denervation-induced untethering of the nNOSμ isoform [27,105], are suggestive of DGC derangement. The enzyme, assembled either in homodimer (μμ) or heterodimer (μβ) conformation, binds both α_1_-syntrophin and the spectrin-like repeats 16–17 of dystrophin by means of its PDZ domain [120,138]. Although nNOS is a cytosolic protein, its docking at sarcolemma requires interaction with the ER chaperone Grp94/gp96, which exists both in lumenal and transmembrane form [28]. In fact, reduced levels of Grp94, as it occurs early in unloading-induced atrophy, jeopardize the targeting of newly synthesized nNOS molecules at sarcolemma and result in increased enzyme concentration in the sarcoplasm [28,29,30], where nNOS fosters both oxidative and nitrosative stresses and FoxO3 activation, as discussed at point 2.2.2 (Figure 1 and Figure 3). The cytosolic chaperone Hsp90 interacts with nNOS, positively affecting its activity and favoring NO production compared to superoxide [120]. Hsp90 and nNOS interaction is increased by muscle training [139], whereas Hsp90 protein levels show only a late reduction after unloading [140]. Interestingly, nNOS, assembled with α-syntrophin, β-dystroglycan, α1-, β-, α2-dystrobrevins, and the carboxyterminal-form of dystrophin Dp71, can localize in the nucleus [76]. Such a complex modulates p53 transcriptional activity in myofibers by means of S-nitrosylation, since the lack of this post-translational modification, which occurs in p53 from old muscles, upregulates atrogene expression [77].

#### 2.3.2. Integrins and Integrin-Associated Signaling

A number of experimental data points to a crucial role of integrins in this context. Integrins are a large family of heterodimeric transmembrane proteins formed by the association of alpha and beta subunits, able to binds to ECM proteins with their extracellular domain, and to the intracellular cytoskeleton through their cytoplasmic regions. Integrins confer more than a physical link between intracellular and extracellular supramolecular structures, in fact they also mediate bidirectional signaling through the plasma membrane, regulating a number of cellular events, including cell migration, adhesion, and proliferation [141]. The α7β1 integrin is highly expressed in skeletal muscle fibers and specifically localizes at costameres and myotendinous junctions [142], where it physically connects the ECM to the sarcomeric contractile apparatus [143]. This location is particularly suitable to sense mechanical stretch. Indeed, tension generated by contraction induces integrin activation and promotes integrin expression, reinforcing cell adhesion to the ECM and enhancing integrin-dependent signaling [144]. Activated integrins, which are connected to the actin cytoskeleton through their intracellular interactors talin and vinculin, cluster along myofiber plasmamembrane and form specialized structures called focal adhesions [145]. The transmembrane proteoglycan syndecan-4 localizes at costameres as well [146,147] and cooperates with integrins in the assembly of focal adhesions and in cytoskeleton organization [148]. Of note, syndecan-4 expression is robustly downregulated after 72 h from denervation [147].

Mechanical strain induces focal adhesion enlargement and stabilization and promotes the recruitment and activation of intracellular signaling molecules. The focal adhesion kinase (FAK) is a non-receptor tyrosine kinase that, upon binding to integrins, undergoes a conformational change that induces its autophosphorylation. This event allows the kinase Src to bind to FAK and to phosphorylate two additional tyrosine residues, mediating its complete activation. FAK activation in skeletal muscle has been described in response to mechanical strain-induced muscle loading, while unloading and denervation decrease FAK expression and phosphorylation [149,150,151,152,153,154,155,156], in a muscle-specific-way [127]. FAK carries on the signal by activating different pathways, such as MAPK and PI3K/Akt pathways, that promote muscle hypertrophic programs [157].

The integrin-linked kinase (ILK) is highly expressed in skeletal muscle, in particular in type II fibers [126], and localizes at myotendinous junctions and costameres, where it binds the cytoplasmic domain of the β1 integrin [158]. ILK recruits actin regulatory proteins, as PINCH, parvin, paxillin, and kindlin [159], impacting on the organization of the actin cytoskeleton. Of note, ILK acts as a connector between the integrin mechanotrasduction apparatus and the insulin-like growth factor I receptor (IGF-IR) signaling platform, coupling mechanical strain to Akt phosphorylation [158,159]. The skeletal muscle-specific inactivation of ILK coding gene alters integrin distribution at the myotendinous junctions, causes a partial detachment of the sarcolemma from the basal membrane, and leads to a progressive muscular dystrophy [158,160]. These features are further aggravated by exercise training, which induces muscle cell necrosis and fibrotic depositions. In response to mechanical strain, phosphorylation of IGF-IR and its downstream effector Akt is impaired in ILK-depleted skeletal muscles [158], suggesting a role for ILK in transducing trophic signals in skeletal muscles. Indeed, ILK is upregulated in muscles in response to exercise [126,161] and it has been involved in transducing hypertrophic stimuli in cardiomyocytes [162,163,164]. Consistently, transcripts for members of both integrin- and ILK-signaling decrease significantly after 5 d-bed rest in both young and old humans [165].

Melusin is a small chaperone protein encoded by the *ITGB1BP2* gene and selectively expressed in the striated muscle tissue, and able to interact with the cytoplasmic domain of integrin β1. From a phylogenetic point of view, melusin has a highly conserved structure, consisting of two cysteine and histidine-rich domains (CHORDS), a domain shared by CHORD proteins and by the co-chaperone protein Sgt1 (CS domain) [166] and a C-terminal Ca^2+^-binding domain [167]. CHORD I-II domains are zinc-binding domains able to mediate the association of melusin to the ATPase domain of HSP90, in its ADP-bound state [168,169,170,171]. The CS domains, structurally similar to α-crystallin and p23 chaperone proteins [172], have been also involved in Hsp90 binding [170,173]. The interaction between melusin and Hsp90 is further favored by the binding of Ca^2+^ to the C-terminal domain, enriched in aspartic and glutamic acid residues [171]. Melusin has been found in complex with FAK and with IQGAP-1, a scaffold protein for the mitogen activated protein kinases c-Raf, MEK1/2 and ERK1/2 [174,175] and with the phosphatidylinositide 3-kinase (PI3K) that activates Akt [176]. In response to mechanical stretch, melusin triggers the integrated activation of ERK1/2 and Akt, promoting cardiomyocyte survival and hypertrophy [177]. In the myocardium, melusin expression is induced by mechanical overload. Indeed, melusin expression levels increase in response to mechanical stress, in conjunction with the compensatory hypertrophic response of the left ventricle [168,178,179,180]. During the subsequent phases of maladaptive remodeling, characterized by chamber dilation, fibrotic tissue deposition and consequent loss of contractile function, melusin expression progressively decreases [178]. Melusin-null mice fail to induce a compensatory hypertrophic response to mechanical overload and rapidly develop a dilated cardiomyopathy, confirming the importance of melusin in regulating cardiomyocyte hypertrophy [181]. The overexpression of melusin protects the myocardium from different challenging conditions, from pressure overload to myocardial infarction and reperfusion injury [178,182,183,184,185], promoting the establishment of a physiological hypertrophic response.

The role of melusin in skeletal muscles has been far less investigated. Melusin starts to be expressed in embryo limbs at 15 d gestation with a peak in newborn muscles. Melusin is highly expressed during secondary myogenesis, when additional myoblasts fuse along the surface of primary myotubes to form secondary myotubes. Melusin expression is maintained in adult skeletal muscles, where it localizes at costameres, and is further induced during muscle regeneration after trauma [167]. Melusin has been found upregulated in muscle from patients affected by limb-girdle muscular dystrophy type 2A (LGMD2A), where it regulates the replacement of the integrin β1A isoform with the β1D isoform, affecting costamere assembly and myotube fusion [186]. We recently identified melusin as a crucial player in muscle atrophy induced by disuse [128]. Muscle unloading induces a drastic and very early drop in melusin expression, well before the onset of muscle atrophy. Indeed, melusin protein level decreases to 50% in rat soleus already after 6 h from tail suspension. A decline in melusin expression has been also noted in the vastus lateralis of patients after eight days of bed rest, suggesting a conserved role in human muscles [128]. Maintenance of physiological levels of melusin expression during unloading by means of cDNA transfection or AAV-9-based gene therapy attenuated muscle atrophy and improved muscle contraction in rats. Of note, forced melusin expression did not affect nNOS activity and FoxO3 nuclear translocation, while clearly dampened Atrogin-1 and MuRF1 expression [128]. The molecular mechanism by which melusin inhibits unloading-induced muscle atrophy does not involve the activation of Akt and ERK pathways, since the co-expression of dominant-negative versions of these kinases did not blunt melusin efficacy in counteracting atrophy [128].

The muscle LIM protein (MLP) is a muscle-specific protein containing two LIM domains involved in protein-protein interactions, able to localize in different cytoplasmic locations, where it binds to different interactors [124,187]. MLP plays crucial structural functions in cardiac muscle, regulating the assembly of supramolecular complexes along the sarcomere and at the Z-disk, as demonstrated by the presence of cytoskeletal disarray in cardiomyocytes and by the development of dilated cardiomyopathy and heart failure in MLP-null mice [188]. Mutations in MLP coding gene are associated with human cardiomyopathy [189] and cause hypertrophic cardiomyopathy and heart failure in mice [190]. Mild abnormalities have been disclosed in skeletal muscle of MLP-null [191] and mutant mice [190], suggesting a role for MLP in maintaining muscle mass and skeletal muscle passive stiffness. Of note, MLP expression levels have been found increased in muscles from mouse models and human patients affected by different types of myopathies [192,193,194]. Recently, MLP has been also involved in promoting autophagosome formation by interacting with LC3, protecting myocytes from apoptosis [195]. MLP localizes at costameres, where it interacts with zyxin [196], β1-spectrin [197] and ILK [198]. Of note, MLP may enter the nucleus and regulate gene transcription by acting as co-activator of transcription factors involved in muscle differentiation such as MyoD, myogenin and MRF4. Interestingly, all these transcription factors appear upregulated in the denervated muscle [199] and MRF4 silencing was demonstrated to abolish denervation-induced muscle atrophy [199]. Transcriptomic data indicate a drop of MLP expression already at 24 h from unloading [68], suggesting a role for MLP in connecting integrin mechanotrasduction to gene expression regulation.

#### 2.3.3. IR/IGF-R

Although IR and IGF-IR are not considered as a canonical component of costameres, the findings demonstrating a physical interaction with several proteins belonging to DGC and integrin complex through plakoglobin [129] or ILK/PINCH [158,159,200], prompt to include these receptors as relevant players. The contribution of IR and its downstream signaling through PI3K-Akt-FoxO to muscle mass regulation is widely acknowledged and of paramount relevance [19]. Indeed, the impairment of IGF-1/insulin signaling induces per se muscle atrophy that can be rescued by triggering the PI3K/Akt/FoxO3 pathway [201,202]. Therefore, this review will focus here only on evidence concerning the interaction of the IR signaling pathway with costamere components. Evidence concerning the early disruption of IR signaling in different contexts of muscle atrophy development will be provided in the next section, together with that one concerning IGF-IR, since both receptors participate in IR signaling pathway [203].

The conductor orchestrating IR and DGC function is represented by plakoglobin (γ-catenin), a desmosomal protein, which in skeletal muscle displays a spot-like distribution in sarcoplasm and sarcolemma, where it colocalizes with dystrophin [204]. Plakoglobin binds to IR and serves as a key component in its interaction with and activation of PI3K and downstream Akt-FoxO signaling. Plakoglobin interaction with PI3K, but not that one with IR, is disrupted by the ubiquitin-ligase Trim 32, which operates on thin filament proteins, Z-band components, and the cytoskeletal costamere-interacting protein desmin. Differently from these targets, Trim32 interaction with plakoglobin does not result in the protein degradation, but in the silencing of PI3K-Akt signaling and in muscle atrophy [204]. A recent investigation showed that plakoglobin participates in a native multimeric assembly, which includes, in addition to IR, DGC components (dystrophin, sarcoglycans, dystroglycans and syntrophins), vinculin, caveolin-1, laminin, and desmin. Precisely, plakoglobulin colocalizes with β-dystroglycan and vinculin, in addition to dystrophin and IR [129]. Proximity ligation assays and domain mapping showed that IR interacts with plakoglobin N-terminus, and β-dystroglycan binds to plakoglobin sites adjacent to this region. Dystrophin binds to plakoglobin central armadillo repeat domain. Such a physical and functional interaction between IR and DGC might mechanistically drive the increased nNOS activation, secondary to Ser1412 phosphorylation, which occurs in skeletal muscle after 10 min of systemic insulin administration [205]. Conversely, reduced plakoglobin protein levels not only affect IR signaling, but also decrease assembly of DCG components and vinculin and promote desmin depolymerization [129].

#### 2.3.4. Na^+^/K^+^ ATPase and Ion Channels

Costamere includes other relevant plasmalemmal components, such as the sodium/potassium pump and the sodium channel [123]. Their inclusion is mediated through ankyrin B and D binding and subsequent anchoring either to spectrin filaments or to the spectrin-like repeats in the dystrophin central region [123,206].

The relevance to consider the sodium/potassium pump is due to its signaling function, in addition to the electrogenic one, in muscle mass regulation (i.e., in cardiac hypertrophy) and in ROS-generation, following its inhibition by ouabain [207]. Partial inhibition of Na^+^/K^+^-ATPase stimulates c-Src- and Ras-dependent signaling, which leads to mitochondrial ATP-sensitive potassium (KATP) channel-related ROS generation. Like ouabain, increases in both exogenous and endogenous ROS can cause conformational changes in Na^+^/K^+^-ATPase and enzyme partial inhibition. Such a signaling cascade involves the α1 subunit of Na^+^/K^+^-ATPase, whereas the α2 subunit, which represents about the 80% of the α subunits in the skeletal muscle, appears to be more involved in electrogenic regulation of muscle contraction, fatigue resistance and exercise performance [208]. Nevertheless, both subunits are upregulated by resistance training in human muscle [209]. Investigations on Na^+^/K^+^-ATPase deregulation in muscle atrophy development are scanty and circumscribed to muscle unloading, where the inhibition of α2 subunits occurs after 6–12 h of unloading, secondary to cholesterol loss from the sarcolemma [210]. A recent study also demonstrated a relevant role of AMPK in the maintenance of α2 subunit activity during a 12-h unloading bout [211].

The voltage-gated sodium channel determines the upstroke as well as the refractory period of the action potential. The density of available sodium channels in the sarcolemma differs between slow and fast fiber populations and greatly influences the firing pattern, which in turn contributes to their phenotypic feature. Both unloading and denervation affect Na^+^ channel expression, but in different manner. The protein levels of the adult skeletal muscle α-subunit isoform of Na^+^-channel encoded by the *SCN4A* gene, transiently increase after one week unloading only in slow-twitch muscles, concomitantly with the change towards a fast-twitch phenotype [212]. Conversely, the increase in total Na^+^-channel mRNA synthesis induced within a week by denervation is accompanied by the appearance of the juvenile/cardiac, tetrodotoxin-resistant Na^+^-channel isoform and of hemichannels (HCs) formed by connexins 39, 43, and 45. Connexin 43 and 45 hemichannels lead to activation of the p65 subunit of NF-κB and up-regulation of pro-inflammatory cytokines (TNF-α and IL-1β) [213]. 

Stretch-activated channels (SACs) are non-specific ion channels that respond to mechanical stress by altering their opening probability and have functional relationships with the DGC and integrins [214,215,216]. SAC opening has been connected to the activation of the Akt/mTOR pro-trophic pathway in skeletal muscle [217]. It has been recently suggested that SACs might undergo functional inactivation during unloading, possibly contributing to atrophy establishment [218]. Among SACs, the stretch-activated and Ca^2+^ permeable TRPC1 channel is expressed in skeletal muscle and interacts with α-1-syntrophin PDZ domain and caveolin-3 [219,220,221,222,223]. This channel has been found to be responsible for anomalous extracellular Ca^2+^ entry in dystrophic muscle fibers [220,222,223]. Downregulation of TRPC1 in adult mouse muscles induces atrophy per se, pointing to a relevant role of this channel in muscle mass regulation [224]. TRPC1 expression is downregulated during muscle unloading and raises again during reloading [224,225] and if TRPC1 expression is suppressed in the reloading phase, muscle regrowth is impaired [224].

## 3. Involvement of Costamere Components in Different Muscle Atrophy Types

The emerging picture from the present literature review indicates a wide variety of potential master regulators of muscle atrophy, whose enrollment during atrophy onset follows the activation of more than a signal transduction pathway and leads to decreased protein synthesis and/or increased protein degradation. Given the differences existing among muscle atrophy phenotypes, a major aim of this review is to enucleate early and relevant players among costamere components and, possibly, hypothetical initiators, presenting available evidence from each research field.

### 3.1. Unloading/Bed Rest/Immobilization

Although all of these three conditions imply reduced muscle load, only immobilization leads to effective loss of muscle activity. During unloading or bed rest, leg gravitational muscles are free to contract, but suffer the absence of body load, which they usually hold in standing position. Indeed, muscle atrophy resulting from each of these conditions shows subtle, yet interesting differences, in muscle contractility, transcriptome and proteome [226]. A number of studies investigated more deeply the effects of short exposure to unloading/inactivity, demonstrating that several events anticipate the morphological evidence of muscle atrophy (Figure 3 and Table 1).

Myosin and actin pre-mRNA transcription decreases already after 24 h-unloading [2], whereas FoxO3, p53, and MAFbx/Atrogin-1 transcript levels quickly increase after exposure to both unloading and immobilization (24 h and 48 h, respectively) [31,68,128,227]. In contrast, time of MuRF-1 mRNA accumulation appears controversial (after 4–7 d of unloading [68,128], 24-h unloading [31] or 48 h-immobilization [227]). FoxO3 upregulation occurs concomitantly with the decrease of Akt activity (24 h-unloading) [128] and the increase in protein ubiquitination and deacetylation (48 h-immobilization) [227]. Loss of active Akt and deacetylation are recognized activators of FoxO3 nuclear translocation [32], the former resulting from blunted IR signaling and the latter from class I HDAC non-histone activity [33].

Another relevant early player involved in FoxO3 activation by unloading is the loss of AMPK activity [19,20,31]. The decrease in AMP levels, secondary to reduced/absent activity in otherwise continuously active gravitational muscles, such as the soleus muscle, leads after 12–24 h of unloading to inactive AMPK accumulation, increased ceramide concentration and p70S6K activation. These detrimental effects, which lead to increased protein synthesis, presumably of key proteolysis regulators, partially relieved after the administration of AICAR (an AMPK activator) [31]. However, AICAR did not blunt MAFbx/Atrogin-1 and MuRF-1 upregulation [31], suggesting that other pathways than phosphorylated p70S6K are involved. Indeed, protein levels of a major target of p70S6K, the Insulin Receptor Substrate 1 (IRS-1), whose Ser-phosphorylation hampers IR signaling and Akt activation, are also significantly decreased after 24 h-unloading [31]. IRS-1 proteostasis appears to be under the control of the ubiquitin-ligase Cbl-b [228], which increases its activity during unloading. Although an early involvement of increased Cbl-b activity has still to be demonstrated, Cbl-b ablation fully counteracted unloading-induced FoxO3 and MAFbx/Atrogin-1 accumulation, muscle mass, and force loss in mice [228].

The early qualitative and quantitative disruption of the IR-signaling pathway apparently follows costamere components disruption, i.e., the decrease in melusin protein levels [128] and the loss of nNOS sarcolemmal activity [30], both of them being detectable 6 h after unloading. Melusin loss is not apparently detrimental for the activity of several of its targets, among which Akt, ERK1/2 and FAK, as shown by melusin replacement together with dominant-negative form of these kinases [128]. Conversely, the redistribution of active/uncoupled nNOS molecules appears to be required upstream FoxO3 nuclear translocation, since decreased nNOS expression, following mRNA interference, or *in vivo* pharmacological inhibition of its enzyme activity, blunted FoxO3 activation [30]. Recent evidence demonstrated the presence of a functional/spatial relationship between DGC and IR, which is lost during fasting (i.e., in a condition leading to muscle atrophy) [129]. The possibility exists that the same “signaling hub” is perturbed by unloading-induced dysfunctions, such as nNOS untethering from DGC, and IRS-1 degradation and/or Ser-phosphorylation occurring roughly simultaneously, and resulting in downstream FoxO3 nuclear translocation. Interestingly, plakoglobin transcripts appear to be upregulated already 1 d after unloading [68], suggesting a compensatory response to early costamere-IR deregulation. Simultaneously with the loss of sarcolemmal nNOS activity, unloading affects the integrin component of costamere. Melusin loss occurs early and before the evidence of atrophy, both in humans (8 d-bed rest) [128] and in rodents (6 h unloading) [128], leading, through still undefined effectors, to atrogene upregulation independently from FoxO3 activation. In fact, melusin replacement attenuated atrophy by means of full downregulation of MAFbx/Atrogin-1 and partial silencing of MuRF-1 and, without affecting FoxO3 nuclear localization and upregulation, which, conversely, appeared paradoxically increased [128]. Indeed, unloading muscle atrophy did not develop after counteracting both melusin loss and nNOS-induced FoxO3 activation. Therefore, unloading-induced muscle atrophy results by the early, parallel and independent involvement of two master regulators: one is FoxO3, activated by the dysregulation at the DGC-IR signaling hub, the other one is the loss of melusin, which primarily involves MAFbx/Atrogin-1 upregulation. Further investigations are necessary to complete the downstream signaling switched on by decreased melusin levels.

Upstream events leading to disruption of both melusin and nNOS await to be fully clarified. Untethering of nNOS from sarcolemma, but not loss of melusin, depends upon increased mitochondrial ROS production occurring after 6 h-unloading [30,128]. Interestingly, ROS upregulate Cbl-b, which deregulates IR signaling [232]. The mechanism involved in increased mitochondrial ROS production after a 6 h-unloading bout is presently unknown. Interestingly, disturbance in lipid distribution and composition of junctional and non-junctional sarcolemma occurs after 6–12 h of unloading [210,231], concomitantly, and probably consequently, to the loss of the Na^+^/K^+^ pump activity, which appears to be regulated in the soleus muscle by both motor activity and sarcolemmal lipid stability (Table 1). Unloading-induced disturbance of sarcolemmal lipids decreases the stiffness of the subsarcolemmal cytoskeleton, mainly composed by non-muscle α-actinin and β- and γ-actin [229]. Indeed, lecithin treatment before 6 h-unloading fully prevented it [230]. Since non-muscle α-actinin binds integrin-related costamere components, such as vinculin and MLP, and γ-actin represents a required component for costamere integrity [124], re-organization of the subsarcolemmal cytoskeleton might be considered as putatively responsible for early costamere and integrin-melusin signaling disruption, eventually leading to disassembly of the DGC-IR, in addition to mitochondrial ROS production.

### 3.2. Denervation/Spinal Cord Injury

Although both nerve crush/transection and spinal cord damage lead to loss of muscle functional responses and induce severe muscle atrophy, there are relevant differences concerning time of appearance of atrophy and type of paralysis [213]. Due to the abundant literature in the field, only data concerning loss of innervation, secondary to nerve transection or compression, or inhibition of neurotransmitter release, will be the object of this review.

As reported for immobilization/unloading, identification of early events requires knowledge of time of appearance of muscle atrophy (Table 2). Available evidence are still controversial: whereas one report indicates 48 h after sciatectomy as the earliest detection time of soleus muscle atrophy, measured by muscle weight normalization to the contralateral innervated one [233], no significant difference in myofiber cross-sectional area was reported at the same stage by another laboratory [234]. Other reports indicated muscle atrophy occurrence three days after denervation, by comparing muscle weight of the denervated muscle either to the contralateral innervated one [235], whose use as a control was recently recognized as a source of potentially flawed results [236], or to age-matched controls [237]. The determination of the earliest evidence of myofiber size loss is crucial to identify upstream events, which might differ from those involved after muscle unloading/immobilization.

A recent trascriptomic analysis of denervated mouse *tibialis anterior* muscle detects atrogene up-regulation only 3 d after sciatectomy [87], in agreement with previous evidence on mouse and rat gastrocnemius muscle [237,238] and at variance with unloading, where atrogene upregulation is detectable already after 24 h [68,128]. Day 3 after sciatectomy also represents the earliest evidence for HDAC4 involvement in denervation atrophy development [235,239]. Although it was suggested that HDAC4 upregulation promoted muscle atrophy by increasing the myogenin-dependent FoxO3 activation [239], recent evidence indicate that the non-histone deacetylase activity of the enzyme has a prominent pro-catabolic effect on different targets, such as molecular chaperones (Hsc70), myofibrillar proteins (myosin heavy chains), and transcription factors (PGC-1α) [240]. Indeed, the acetylated (inhibited) FoxO3 form is strongly reduced 3 d after denervation [32]. Strikingly, HDAC4 nuclear import to exert histone-deacetylase activity requires the activation of Akt-mTORC1 signaling, i.e., the main inhibitor of FoxO gene family transcriptional activity [235]. Such a paradoxical context appears, however, necessary to allow HDAC4-dependent synaptic gene expression and endplate maintenance in the denervated muscle.

Recent investigations provided a major advance in knowledge of early events in the development of denervation-induced muscle atrophy by analyzing muscle transcriptome at different times after denervation, from less than 0.5 h to 28 d. Major findings were the up-regulation of genes involved in the oxidative stress and inflammatory responses within 0.5–24 h after denervation [59,87]. Inflammation contributes to muscle atrophy development in several contexts [241]. Its early activation after denervation might be related to nerve injury and stump degeneration [242] and/or to the early increase in muscle levels of oxidative stress, as suggested by the upregulation of cytochrome P450 and monooxygenase transcripts, together with others involved in the anti-oxidant defense, about 3 h after denervation [87,243]. Activation of NF-κB and pro-inflammatory cytokines, secondary to de novo expression of connexins after one-week denervation, also contributes to muscle atrophy [213].

The actual knowledge of oxidative stress source(s) in the denervated muscle remains elusive. Transcripts of both PCG-1α and β, the regulators of mitochondrial proliferation, dramatically decrease after 24 h-denervation [237], suggesting early impairment of mitochondrial pool renewal. Monoamine oxidase A transcript and protein content was found to be increased between 3–24 h after denervation [87,244]. However, ROS production from mitochondria apparently increased later than 48-h denervation [103,104,244], possibly in concomitance with increased mitochondria misplacement at A band [245]. The possibility exists that other ROS sources do contribute to a very early increase in oxidative stress. Deregulation of intracellular Ca^2+^ levels might represent a possible initiator. Interestingly, changes in Ca^2+^ release and uptake from the SR lead to transiently reduced levels of stored Ca^2+^ as early as after 48 h-denervation, and, conversely, to their increase after 7-d denervation [234]. Mitochondrial Ca^2+^ uptake appears to be reduced after 3-d denervation [245]. We tentatively speculate that the earlier deregulation of Ca^2+^ cycling follows the transitory increased expression (between 0.5 and 6 h after denervation) of genes involved in ion release and binding in the cytosol [87]. The biological significance of this transient increase in cytosolic calcium levels remains obscure. In addition to excitation-contraction coupling, both the sarcolemmal calcium channel/dihydropyridine receptor and the SR calcium channel/RYR1 play a relevant regulatory role in NMJ development and destabilization [246]. Furthermore, through Ca^2+^-calmodulin, Ca^2+^ transients might stimulate nNOS coupled and uncoupled activity, fostering both ROS and RNS accumulation [120]. Calcium ions also increase phospholipase A2 (PLA2) activity [247]. Phospholipase A2 de-esterifies membrane phospholipids, which can promote enzymatic (i.e., via lipo-hydroperoxidases) and non-enzymatic peroxidation of bis-allylic unsaturated lipids and activate NADPH-oxidases [248]. Interestingly, muscle lipo-hydroperoxides increase between 2–4 d of denervation consequently to cytosolic PLA2 activation [59]. Protein levels of peroxiredoxin 6, which has potential PLA2 activity, also increase after 3 d-denervation [244]. Calcium depletion from SR stores might trigger an ER-stress response in denervated myofibers [21]; increased expression of the transcription factor ATF4 was observed within 2–4 d after denervation [59]. Increase in sarcoplasmic calcium levels also affects costamere components. By means of calpain activation, desmin is proteolyzed after 4 d-denervation [249]. Interestingly, proteolysis followed desmin interaction with and phosphorylation by GSK3-β and Ca^2+^-calmodulin kinase, and protein ubiquitination 3 d after denervation [249].

Denervation-induced dysregulation of costamere components may represent an early target. After 3-d denervation, the levels of syndecan-4, a cell surface heparan-sulfate proteoglycan co-operating with integrins and involved in PKC signaling, are reduced [147]. Accordingly, transcript downregulation of focal adhesion components and ECM receptors was reported after 6-h denervation [87]. Interestingly, some nPKC forms are selectively recruited in the muscle membrane fraction as early as 24-h denervation [250]. Available evidence concerning gene expression dysregulation of costamere components, among which melusin [251], and nNOS redistribution to sarcoplasm [27,110,126,252] were collected only after the establishment of denervation atrophy (i.e., after 7–14 d). A recent report identified perlecan-α-dystroglycan interaction as a major site responsible for nNOS untethering from DGC as early as 4 d after denervation [253]. Indeed, no reduction in nNOS sarcolemmal localization and attenuated muscle atrophy were observed in denervated gastrocnemius of perlecan KO mice [253]. Preliminary data obtained in our laboratory reveal that the nNOS-interacting Grp94/gp96 chaperone and melusin are involved very early after denervation, since the levels of these two proteins significantly decreased from one day after denervation (M. Brancaccio and L. Gorza, unpublished observations). Early denervation-induced derangement of costamere proteins has still to be fully investigated, but several studies focused on IR signaling, a major component of the costamere signaling hub [129]. The occurrence of insulin resistance (decreased glucose uptake) appears as early as 24 h after denervation, although without alteration in the IR ability to bind insulin or transfer downstream signaling to PI3K and Akt [254], and it is followed by a marked GLUT-4 downregulation 3 d after denervation [255].

Eventually, components of the DGC are also relevantly involved in the stabilization of the NMJ, whose disruption is evident in most myofibers 3 h after denervation. Similarly to unloading, denervation perturbs the membrane lipid composition by dispersing the cholesterol-rich lipid membrane domains where post-synaptic proteins reside, among which the acetylcholine receptors, and some DGC components [256]. Interestingly, the pro-growth transcription factor YAP accumulates in adult muscle fibers at the membrane near the NMJ. The DGC, together with agrin, an essential regulator of NMJ stability, is involved in YAP inhibition through Hippo signaling [22]. Denervation results in accumulation of the Hippo-pathway kinase MST1 and increased YAP abundance as early as 6 h after sciatectomy. Sustained increase in YAP transcription, phosphorylation at Ser112, and total protein occurred along 14 d post-denervation, concomitantly with the protein localization in myonuclei [22].

### 3.3. Cachexia

A common mechanism inducing cachexia in patients affected with cancer and different pathological conditions is represented by circulating factors, which may originate from different tissues and include cytokines, extracellular vesicles, hormones, and growth factors. These systemic mediators may act directly on skeletal muscle cells or indirectly by inducing a metabolic rewiring in other tissues that will subsequently drive skeletal muscle wasting [11,257].

Pro-inflammatory cytokines bind to surface receptors on muscle cells and trigger signaling pathways that cooperate to induce and activate ubiquitin ligases and autophagy regulators, promoting protein catabolism. Indeed, TNF-α, TWEAK, and IL-1 activate NF-κB, known to induce MuRF1 and to promote protein catabolism. Interferon-γ and IL-6 activate the STAT3 pathway that induces cytokine production and cooperates with NF-κB in promoting atrophy [257,258]. MAP kinase signaling cascades involving ERK, p38 and JNK have also been involved in inducing cachexia in different studies [259,260,261].

Myostatin family proteins have been reported to play a crucial role in cancer cachexia. By binding the surface receptor ActRIIB, they activate Smad2/3 signaling pathway, promote FoxO3 activity and the subsequent transcription of Atrogin-1 and MuRF1. The inhibition of ActRIIB cascade reverts muscle loss and improves survival in different cancer mouse models [262,263,264]. In line, the inhibition of the Smad2/3 signaling by overexpressing the Smad inhibitory protein Smad7 prevents muscle cachexia in cancer bearing mice [265].

Systemic stress signals mediated by chaperone proteins are able to trigger skeletal muscle atrophy. Stressed cells, as cancer cells or cardiomyocytes subjected to mechanical overload, activate unconventional mechanisms of protein secretion [266]. The chaperone proteins Hsp70 and Hsp90 are released in a soluble form or on the surface of extracellular vesicles and, once reached skeletal muscle fibers, bind to Toll-like receptor (TLR) 4 and trigger the p38 pathway, inducing muscle wasting [267].

On the other hand, the attenuation of trophic pathways as IGF-1 and insulin mediated signals on skeletal muscle fibers contributes to muscle cachexia too. IGF-1 and insulin activate, through PI3K, the serine threonine kinase Akt, a potent inhibitor of FoxO3 [268,269,270]. In cachectic rodents and patients, the expression of IGF-1 in muscles and in the circulation decreases [271,272,273] In one study, IGF-1 administration has been shown to reduce weight loss and improve survival in cancer-bearing rodents [274]. Of note, cachectic cancer patients suffer of insulin resistance and administration of insulin [275] or insulin sensitizers [276] may reduce muscle wasting [137,277]. It has been recently demonstrated that plakoglobin connects DGC to IR and the disruption of this supramolecular complex impairs insulin signaling and induces muscle atrophy [129], suggesting that insulin resistance may depend on the alterations of costamere integrity. The forced reduction of plakoglobin expression levels in muscle results in impaired PI3K/Akt signaling and muscle atrophy [204]. Interestingly, it has been shown that the plasma membrane of cachectic muscle fibers show an irregular morphology, due to the decrease in dystrophin expression by post-translational mechanisms, the concomitant upregulation of utrophin, and the aberrant glycosylation of β-dystroglycan and β-sarcoglycan [136]. Destabilization of the DGC may therefore represent a new mechanism through which cachectic factors induces muscle loss.

### 3.4. Sarcopenia

Sarcopenia development has been attributed to multiple mechanisms, among which a major role has been hypothesized for the increase in both oxidative and nitrosative stresses [91,278], the loss of innervation [7,279], and the decreased regenerative potential of muscle stem cells [81,280].

ROS accumulation by dysfunctional mitochondria, consequent to impaired removal by autophagy [281], elicits senescence and the onset of age-related diseases. Increased protein carbonyl adducts characterize old skeletal muscle mitochondria, independently of sarcopenia [282]. The possibility that partial muscle denervation, which accompanies muscle aging, would increase ROS production in the remaining innervated fibers, and, therefore, promote sarcopenia, was confirmed by the evidence of generalized myofiber atrophy and increased mitochondrial ROS levels [104].

To the aged muscle dysfunctions contributes the nitrosative stress, secondary to increased NO production and nNOS/eNOS protein levels, which accumulate in the sarcoplasm [91,283,284,285]. However, decreased nNOS enzyme level and activity, and targeted S-nitrosylation in sarcopenic muscle have been reported too [286,287]. We cannot therefore exclude that such a controversial body of evidence reflects species- and muscle-specific differences. The failure in S-nitrosylation fosters both atrogene expression and myofibrillolysis [77,287]. The reduced S-nitrosylation of p53, secondary to a defective shuttle of nNOS to the nucleoskeleton, results in MuRF-1 gene upregulation [77], which is among the few atrogenes involved in sarcopenia [7,26]. In fact, FoxO3 activation appears modest in aging muscles [25], whereas p53 protein level is higher compared to the adult one [64]. Lack of calpain S-nitrosylation leads to increased proteolysis of myofibrillar proteins (myosin and troponins) and the intermediate filament scaffold (desmin), and to a minor proteolytic modification of α-actinin, which could disrupt interactions between thin filaments and the Z-disk [287].

The accumulation of oxidatively modified proteins and protein aggregates in the presence of myofibrillolysis points to a dysregulation of the intracellular proteolytic systems. Proteasomal activity is declining in the aged muscle, whereas the autophagy-lysosomal system shows a muscle-specific derangement, being severely impaired in fast-twitch muscles, whereas only mildly reduced in slow-twitch ones [90].

Increased remodeling of muscle connective tissue and availability of myostatin have been also considered as possible initiators of sarcopenia [53]. However, the variable results concerning serum and muscle myostatin levels and loss of muscle mass in humans apparently rule out a major role for this signaling pathway [25], despite the evidence of sarcopenia decrease following myostatin inhibition in animal investigations [34]. In fact, myostatin KO or administration of anti-myostatin antibodies attenuated muscle fiber atrophy, enhanced muscle functional capacity, and reduced apoptosis in skeletal muscles of aging mice [288]. On the other hand, myostatin negatively regulates satellite cell proliferation and commitment to differentiation, reducing the recruitment of satellite cells [81]. In addition, increased levels of p53 may decrease satellite cell commitment by binding directly to the myogenin promoter and repressing transcription [71].

Costamere composition is affected variably by aging. Changes at this level or at costamere anchoring to myofibrils may lead to impaired force transfer and cause the loss in muscle strength occurring in the aged muscle, especially in the presence of a light reduction in muscle mass [15]. The major change concerns the loss of dystrophin, which, in the very old rat, occurs in a muscle-specific manner, before appearance of myofiber atrophy, and independently from gene expression [289]. In old rat *plantaris*, discontinuous membrane expression of dystrophin and α-syntrophin are accompanied by reduced sarcolemmal nNOS localization [285]. In contrast, in murine old soleus, the sarcolemmal distribution of the active enzyme remains largely detectable, showing increased accumulation at discrete foci (L.Gorza, unpublished observation), despite the reduction in total nNOS protein levels (Samengo et al. 2012; L. Gorza unpublished observations). Reduced dystrophin levels decrease lateral force transmission, leading to sarcomere and NMJ instability and subsequent contraction-induced injury [289,290], despite the presence of increased expression of other DGC and costamere components [285,289]. Although exposure of hindlimb muscles of aged rats to unloading did not reduce further dystrophin protein levels, the compensatory increase of DGC and costamere components does not prevent the muscle membrane damage and regeneration following reloading [135].

Desmin participates in the DGC compensatory response of old muscles, by increasing protein levels in a muscle-specific way [289,291,292]. Strikingly, desmin phosphorylation levels are increased in the aging muscles [291], suggesting ongoing depolymerization of desmin filaments [249]. In addition to link adjacent myofibrils to each other at the periphery of sarcomere Z-discs and M-bands, desmin filaments anchor them to the sarcolemma, via plectin and costameres, as well as to the mitochondria and the nucleus, playing a major role in the maintenance of sarcomere alignment [124]. A two-dimensional finite-element model, developed to investigate the mechanical contribution of desmin in a fixed-end contraction, predicted a higher maximum stress production when desmin filaments concentrate in the subsarcolemma, compared to the fiber center [293]. Both increased folding of sarcolemma, and loss and disorganization of subsarcolemmal myofibrils occur in aged myofibers, favoring desmin accumulation in intermyofibrillar and subsarcolemmal spaces, as suggested by the coarser pattern of labelling with anti-desmin antibodies [294]. The possibility exists that the increased desmin accumulation would contribute to the age-dependent increase of muscle stiffness, which is relevant to the preservation of eccentric force generation in the elderly [295].

Despite the presence of signs of DGC derangement, IR signaling does not appear to be disrupted during aging [25], such as it occurs in the absence of dystrophin expression [129]. Such a feature deserves further investigations, taking into account the body of evidence concerning the muscle-specific loss of dystrophin and/or the fiber-type specific responses to aging [1,135,285,289].

## 4. Conclusions

Although the investigation on early events leading to muscle atrophy is still at its beginning, the possibility that more than one master regulator is required and involved in the atrophic process is supported by increasing experimental evidence. The costamere appears as the least investigated muscle compartment during muscle atrophy development, compared to myofibril proteins or mitochondria. Nevertheless, recent investigations indicate this site as the most crucial one, because of the regulatory coupling among IR/IGFR and DGC/integrin, and the most responsive one, for the early deregulation of its components involved in nitrosative/oxidative stress and signaling regulation, such as nNOS and melusin in unloading-induced muscle atrophy.

Further studies are therefore required to determine the contribution of costamere deregulation to the development of other muscle atrophy types, although a few available evidences are already suggestive of an early involvement of some of its components.

## Figures and Tables

**Table 1:** Temporal sequence of events induced by unloading/inactivity in rodent hindlimb muscles before the appearance of myofiber atrophy.

Time	Localization	Event	Consequences	Reference
6 h	costamere	loss of melusin	increased FoxO3-independent transcription of MAFbx-Atrogin-1	[128]
		redistribution of non-muscle α-actinin and β- and γ- actin	subsarcolemmal cytoskeleton reorganization	[229,230]
		loss of active sarcolemmal nNOS	FoxO3 nuclear translocation	[30]
		decreased sarcolemmal NO production	AMPK inactivity	[20]
	sarcoplasm	increased accumulation of active/uncoupled nNOS	increased NO/ROS production	[30]
	mitochondria	ROS production	loss of active sarcolemmal nNOS	[30]
		ROS production	covalent S-S tropomyosin species	[30]
	sarcolemma	loss of cholesterol	membrane instability loss of Na/K pump activity	[210,231]
		inhibition of _2_subunit of Na/K pump	membrane instability	[231]
	nucleus	increased FoxO3 localization	transcriptional activity	[30]
		decreased nNOS transcription	decreased nNOS protein	[30]
12 h	sarcoplasm	AMPK inactivity	p70S6K activation loss of Na/K pump activity	[20,211]
		ceramide accumulation	sarcolemma instability	[210]
		increased phospo-p70S6K	pSer-IRS-1	[20,31]
24 h	sarcolemma/costamere	decreased levels of IRS-1	impaired Akt activation	[31]
		decreased integrin signaling activation	impaired Akt, ERK1/2 activation	[31,128]
	nucleus	increased FoxO3 transcription	increased protein levels and atrogene upregulation	[31,68,128,227]
		myosin gene down-regulation	fiber type transition	[2]
		multiple transcriptional changes	phenotype change	[68]
	sarcoplasm/nucleus	increased p53 protein levels	MuRF-1 and p21 upregulation	[57]
48 h	SR/ER	decreased Grp94/gp96 levels	decreased nNOS delivery to sarcolemma	[30]
	sarcoplasm	increased protein ubiquitination and deacetylation by HDAC1	increased protein catabolism, FoxO3 activation	[32,227]
		increased Bax/Bcl2 ratio; mitochondrial release of AIF; caspase -3 and caspase-8 activation	increased myonuclei apoptosis	[73]
		reduced myofiber cross-sectional area	earliest morphological evidence of muscle atrophy	[30]

**Table 2:** Temporal sequence of events induced by denervation in rodent hindlimb muscles before the appearance of myofiber atrophy.

Time	Localization	Event	Consequences	Reference
0.5–6 h	nucleus	increased gene transcription for: calcium-release channels and calcium-binding proteins oxidative stress YAP	increased sarcoplasmic calcium binding; increased oxidative stress; increased YAP	[59,80,87]
		decreased gene transcription for: focal adhesion and extracellular receptors	reduced mechanotransduction	[87]
3 h	sarcolemma	neuromuscolar junction disruption	AChR clustering	[256]
6 h	sarcoplasm	increased protein levels of Hippo kinase MST1 and YAP	reduced YAP signaling	[22]
	nucleus	YAP localization	increased YAP signaling	[22]
24 h	sarcolemma/costamere	inhibition of IR signaling without Akt inhibition	decreased glucose uptake	[254]
	sarcoplasm	increased Monoamine oxidase A	oxidative stress	[87,244]
		DAG-sensitive nPKC binding to intracellular membranes	impaired insulin-stimulated glycogen synthesis	[250]
	nucleus	PCG- and - genes down-regulation	reduced mitochondriogenesis	[237]
		up-regulation of pro-inflammatory genes	activation of NF- B pathway	[59,87,213]
48 h	SR	decreased Ca^2+^ uptake	reduced levels of stored Ca^2+^	[234]
		reduced levels of stored Ca^2+^	ER stress response	[21]
48–72 h	sarcoplasm	increased protein ubiquitination and deacetylation by HDAC4	increased protein catabolism, FoxO3 activation	[32,227,240]
		increased muscle lipo-hydroperoxides by phospholipase A2	oxidative stress via NADPH-oxidase	[59,248]
		reduced muscle mass	earliest evidence of muscle atrophy	[233,235,237]
	nucleus	increased ATF4 expression	ER stress response	[59]
72 h	costamere	active GSK3- and Ca^2+^-calmodulin kinase	desmin phosphorylation/ubiquitination	[249]
		reduced syndecan 4	reduced integrin signalling	[147]
	mitochondria	misplacement at A band	reduced connection with triads and Ca^2+^ uptake	[245]
		increased ROS production and expression of mitochondrial anti-oxidant enzymes	oxidative stress	[103,104,244]
	nucleus	FoxO3, HDAC4 upregulation	atrogene up-regulation	[87,235,237,238,239]
		GLUT-4 down-regulation	reduced glucose import	[255]
	sarcoplasm	increased peroxiredoxin 6	increased phospholipase A2 activity	[244]

## Data Availability

No new data, except for preliminary ones, were created or analyzed in this study. Data sharing is not applicable to this article.

## Abbreviations

4-PBA	4-phenyl butyric acid
AAV-9	Adeno-associated virus serotype 9
Ac	Acetylation
ActRIIB	Activin receptor type IIB
AICAR	5-amino-imidazole-4-carboxamide ribonucleotide (AMPK activator)
Akt	Protein kinase B
AMP	Adenosine monophosphate
AMPK	AMP-activated protein kinase
ATF4	Activating transcription factor 4
ATP	Adenosine triphosphate
BAFFR	Tumor necrosis factor receptor superfamily member 13C
cAMP	Cyclic adenosine monophosphate
Cbl-b	Casitas B-lineage lymphoma-b ubiquitin ligase
CD40	Cluster of differentiation 40
CHORDS	Cysteine and Histidine-Rich Domains
c-Raf	RAF proto-oncogene serine/threonine-protein kinase
c-Rel	Proto-oncogene c-Rel
CS	CHORD and Sgt1 domain
DGC	Dystrophin glycoprotein complex
ECM	Extracellular matrix
ERK1/2	Extracellular signal-regulated kinase 1/2
FAK	Focal adhesion kinase
FOXO1/3/4	Forkhead box O1/3/4
GLUT-4	Glucose transporter 4
Grp94/gp96	Glucose-regulated protein 94/glycoprotein 96
GSK-3β	Glycogen synthase kinase-3β
HDAC1/4	Histone deacetylase 1/4
HER2	Receptor tyrosine-protein kinase erbB-2
Hsp90	Heat shock protein 90
IGF1R	Insulin-like growth factor 1 receptor
IGF-I and –II	Insulin-like growth factor I and II
IKKα/β	Inhibitor of nuclear factor kappa-B kinase subunit α/β
IL-1	Interleukin 1
ILK	Integrin-linked kinase
IQGAP-1	IQ Motif Containing GTPase Activating Protein 1
IR	Insulin receptor
IRS-1	Insulin receptor substrate-1
*ITGB1BP2*	*Integrin beta-1-binding protein 2*
IκB	Inhibitor of nuclear factor kappa B
JNK	c-jun N-terminal kinase
LGMD2A	Limb-girdle muscular dystrophy type 2A
LTβR	Lymphotoxin-β receptor
MAFbx	Muscle atrophy F-box ubiquitin ligase
MAPK	Mitogen-activated protein kinase
MEK1/2	Dual specificity mitogen-activated protein kinase kinase 1/2
MLP	Muscle LIM protein
MRF4	Myogenic regulatory factor 4
MST1	Macrophage stimulating 1
mTORC1	Mammalian/mechanistic target of rapamycin
MuRF-1	Muscle RING finger—1 ubiquitin ligase
*MyoD*	Gene coding for Myoblast determination protein 1
NADPH	Nicotinamide adenine dinucleotide phosphate
NF-κB	Nuclear factor kappa-light-chain-enhancer of activated B cells
NF-κB1	Nuclear factor NF-kappa-B p105 subunit
NF-κB2	Nuclear factor NF-kappa-B p100 subunit
NIK	NF-κB inducing kinase
NMJ	Neuromuscular junction
nNOS	Neuronal nitric oxide synthase
NO	Nitric oxide
p38 MAPK	p38 mitogen-activated protein kinase
p70S6K	Ribosomal protein S6 kinase p70
PCG-1α and β	Peroxisome proliferator-activated receptor-gamma coactivator 1α and β
PDZ domain	PSD-95/Dlg/ZO-1 domain
PI3K	Phosphoinositide 3-kinase
PINCH	Particularly interesting new Cys-His protein 1
PKC	Protein kinase C
PLA2	Phospholipase A2
*PW1*	Gene coding for Paternally-expressed gene 3 protein
RANK	Tumor necrosis factor receptor superfamily member 11A
RelA/p65	Transcription factor p65
RelB	Transcription factor RelB
ROS	Reactive oxygen species
RYR1	Ryanodine receptor 1
SAC	Stretch-activated ion channel
*SCN4A*	Gene coding for Sodium channel protein type 4 subunit alpha
Smad	Small mother against decapentaplegic
SR	Sarcoplasmic reticulum
TAZ	Transcriptional coactivator with PDZ-binding motif
TLR	Toll-like receptor
TNFR	Tumor necrosis factor receptor
TNF-α	Tumor necrosis factor a
Trim 32	Tripartite motif-containing protein 32
TRPC1	Transient receptor potential channel 1
TWEAK	TNF-related weak inducer of apoptosis
YAP	Yes-associated protein
